# Supplementary material for: Supporting mental well-being of healthcare workers using a mobile app: A mixed-methods feasibility study
Source: PLoS One. 2026 Jan 16;21(1):e0341055. doi: 10.1371/journal.pone.0341055 (PMC12810850; doi:10.1371/journal.pone.0341055)
Supplement: S1 Table — (DOCX) [file pone.0341055.s001.docx]

**S1 Table.** All mental wellbeing-related questions and possible answers in the mobile app

| **No** | **Questions** | **Answer options** | **Follow up question** | **Answer options** |
| --- | --- | --- | --- | --- |
| 1 | How are you today? | Hopeless |  |  |
|  |  | Low |  |  |
|  |  | Okay |  |  |
|  |  | Good |  |  |
|  |  | Great |  |  |
| 2 | How many meals did you eat today? | 0 |  |  |
|  |  | 1 |  |  |
|  |  | 2 |  |  |
|  |  | 3 |  |  |
|  |  | 4+ |  |  |
| 3 | How many hours did you sleep last night? | 0-3 |  |  |
|  |  | 4--6 |  |  |
|  |  | 7--9 |  |  |
|  |  | 10+ |  |  |
| 4 | Did you exercise today? | Not at all |  |  |
|  |  | Somewhat active |  |  |
|  |  | Fairly active |  |  |
|  |  | Very active |  |  |
|  |  | Excessively active |  |  |
| 5 | Did you work today? | Yes | How many hours? | 1-4 hours  5-9 hours  10+ |
|  |  | No |  |  |
| 6 | How many caffeinated drinks today? | 0 |  |  |
|  |  | 1--3 |  |  |
|  |  | 4--6 |  |  |
|  |  | 7--9 |  |  |
|  |  | 10+ |  |  |
| 7 | Have you felt work challenges today? | No |  |  |
|  |  | Yes, I can deal with it. |  |  |
|  |  | Yes, I can't deal with it. |  |  |
| 8 | Have you felt low energy today? | No |  |  |
|  |  | Yes, I can deal with it. |  |  |
|  |  | Yes, I can't deal with it. |  |  |
| 9 | Have you felt general worry today? | No |  |  |
|  |  | Yes, I can deal with it. |  |  |
|  |  | Yes, I can't deal with it. |  |  |
| 10 | Have you felt anxiety today? | No |  |  |
|  |  | Yes, I can deal with it. |  |  |
|  |  | Yes, I can't deal with it. |  |  |
| 11 | Have you felt unable to focus today? | No |  |  |
|  |  | Yes, I can deal with it. |  |  |
|  |  | Yes, I can't deal with it. |  |  |
| 12 | How many alcoholic drinks today? | 0 |  |  |
|  |  | 1--3 |  |  |
|  |  | 4--6 |  |  |
|  |  | 6+ |  |  |
| 13 | Have you felt relationship challenges today? | No |  |  |
|  |  | Yes, I can deal with it. |  |  |
|  |  | Yes, I can't deal with it. |  |  |
| 14 | Did you have your period today? | No |  |  |
|  |  | Yes, I can deal with it. |  |  |
|  |  | Yes, I can't deal with it. |  |  |
| 15 | Did you experience menopause symptoms today? | No |  |  |
|  |  | Yes, I can deal with it. |  |  |
|  |  | Yes, I can't deal with it. |  |  |
| 16 | Have you felt family challenges today? | No |  |  |
|  |  | Yes, I can deal with it. |  |  |
|  |  | Yes, I can't deal with it. |  |  |
| 17 | Have you felt physical pain today? | No |  |  |
|  |  | Yes, I can deal with it. |  |  |
|  |  | Yes, I can't deal with it. |  |  |
| 18 | Have you taken your medicine today? | No |  |  |
|  |  | Yes |  |  |
| 19 | How much did you smoke/vape today? | 0 |  |  |
|  |  | 1--3 |  |  |
|  |  | 4--6 |  |  |
|  |  | 6+ |  |  |
| 20 | Have you felt lonely? | No |  |  |
|  |  | Yes, I can deal with it. |  |  |
|  |  | Yes, I can't deal with it. |  |  |
| 21 | Have you felt financial challenges today? | No |  |  |
|  |  | Yes, I can deal with it. |  |  |
|  |  | Yes, I can't deal with it. |  |  |
| 22 | Have you felt irritable today? | No |  |  |
|  |  | Yes, I can deal with it. |  |  |
|  |  | Yes, I can't deal with it. |  |  |
| 23 | Have you felt anger today? | No |  |  |
|  |  | Yes, I can deal with it. |  |  |
|  |  | Yes, I can't deal with it. |  |  |
| 24 | Have you felt agitation today? | No |  |  |
|  |  | Yes, I can deal with it. |  |  |
|  |  | Yes, I can't deal with it. |  |  |
| 25 | Have you felt tearful? | No |  |  |
|  |  | Yes, I can deal with it. |  |  |
|  |  | Yes, I can't deal with it. |  |  |
| 26 | Have you felt withdrawn today? | No |  |  |
|  |  | Yes, I can deal with it. |  |  |
|  |  | Yes, I can't deal with it. |  |  |
| 27 | Did you struggle with your disability? | No |  |  |
|  |  | Yes, I can deal with it. |  |  |
|  |  | Yes, I can't deal with it. |  |  |
| 28 | Have you felt hyper? | No |  |  |
|  |  | Yes, I can deal with it. |  |  |
|  |  | Yes, I can't deal with it. |  |  |
