# Supplementary material for: Supporting mental well-being of healthcare workers using a mobile app: A mixed-methods feasibility study
Source: PLoS One. 2026 Jan 16;21(1):e0341055. doi: 10.1371/journal.pone.0341055 (PMC12810850; doi:10.1371/journal.pone.0341055)
Supplement: S3 Table — (DOCX) [file pone.0341055.s003.docx]

**S3 Table.** COREQ-32 checklist

| **No** | **Item** | **Guide questions/description** | **Answer** |
| --- | --- | --- | --- |
| **Domain 1: Research team and reflexivity** |  |  |  |
| Personal Characteristics |  |  |  |
| 1. | Interviewer/facilitator | Which author/s conducted the interview or focus group? | Author (MY) |
| 2. | Credentials | What were the researcher's credentials? E.g. PhD, MD | PhD student, Nursing BSc, MSc |
| 3. | Occupation | What was their occupation at the time of the study? | PhD student and Research Associate in Health Sciences |
| 4. | Gender | Was the researcher male or female? | Male |
| 5. | Experience and training | What experience or training did the researcher have? | Trained in qualitative research methods |
| **Relationship with participants** |  |  |  |
| 6. | Relationship established | Was a relationship established prior to study commencement? | No |
| 7. | Participant knowledge of the interviewer | What did the participants know about the researcher? e.g. personal goals, reasons for doing the research | PhD student |
| 8. | Interviewer characteristics | What characteristics were reported about the interviewer/facilitator? e.g. Bias, assumptions, reasons, and interests in the research topic | Gender  Occupation |
| **Domain 2: study design** |  |  |  |
| Theoretical framework |  |  |  |
| 9. | Methodological orientation and Theory | What methodological orientation was stated to underpin the study? e.g. grounded theory, discourse analysis, ethnography, phenomenology, content analysis | Braun and Clarke’s thematic approach |
| Participant selection |  |  |  |
| 10. | Sampling | How were participants selected? e.g. purposive, convenience, consecutive, snowball | Convenience sampling |
| 11. | Method of approach | How were participants approached? e.g. face-to-face, telephone, mail, email | Online through social media and e-mail |
| 12. | Sample size | How many participants were in the study? | 13 of 16 who started the intervention |
| 13. | Non-participation | How many people refused to participate or dropped out? Reasons? | 3  Lost to follow up (1)  Losing interest (1)  Traffic accident (1) |
| Setting |  |  |  |
| 14. | Setting of data collection | Where was the data collected? e.g. home, clinic, workplace | Based on participant’s online availability (not questioned) |
| 15. | Presence of non-participants | Was anyone else present besides the participants and researchers? | No |
| 16. | Description of sample | What are the important characteristics of the sample? e.g. demographic data, date | n/a |
| Data collection |  |  |  |
| 17. | Interview guide | Were questions, prompts, guides provided by the authors? Was it pilot tested? | Yes |
| 18. | Repeat interviews | Were repeat interviews carried out? If yes, how many? | No |
| 19. | Audio/visual recording | Did the research use audio or visual recording to collect the data? | Video recording by the participant’s consent |
| 20. | Field notes | Were field notes made during and/or after the interview or focus group? | No |
| 21. | Duration | What was the duration of the interviews or focus group? | lasting 11–37 minutes (mean: 19 minutes) |
| 22. | Data saturation | Was data saturation discussed? | Aimed to recruit all participants from the intervention group but recruited 13 |
| 23. | Transcripts returned | Were transcripts returned to participants for comment and/or correction? | No |
| **Domain 3: analysis and findings** |  |  |  |
| Data analysis |  |  |  |
| 24. | Number of data coders | How many data coders coded the data? | One (MY) |
| 25. | Description of the coding tree | Did authors provide a description of the coding tree? | Yes |
| 26. | Derivation of themes | Were themes identified in advance or derived from the data? | Derived from the data |
| 27. | Software | What software, if applicable, was used to manage the data? | NVivo v12 |
| 28. | Participant checking | Did participants provide feedback on the findings? | No |
| Reporting |  |  |  |
| 29. | Quotations presented | Were participant quotations presented to illustrate the themes / findings? Was each quotation identified? e.g. participant number | Yes (page 18-24) |
| 30. | Data and findings consistent | Was there consistency between the data presented and the findings? | Yes (page 18-24) |
| 31. | Clarity of major themes | Were major themes clearly presented in the findings? | Yes (page 18-24) |
| 32. | Clarity of minor themes | Is there a description of diverse cases or discussion of minor themes? | Yes (page 18-24) |
