# Supplementary material for: Supporting mental well-being of healthcare workers using a mobile app: A mixed-methods feasibility study
Source: PLoS One. 2026 Jan 16;21(1):e0341055. doi: 10.1371/journal.pone.0341055 (PMC12810850; doi:10.1371/journal.pone.0341055)
Supplement: S6 Table — (DOCX) [file pone.0341055.s006.docx]

**S6 Table.** Themes and sample quotes

| Themes | Sample quotes |
| --- | --- |
| Usefulness |  |
| - Impact on behaviours | “Another thing maybe I can say my caffeine intake. I am drinking lots of coffee, but I try to decrease my caffeine intake. I try to drink one, two, or three coffees.” Participant 16, Healthcare student, Woman.  “I think it was useful in terms of like sleep, like monitoring your sleep and stuff because that’s one thing that I’ve been trying to get more of, so that was useful.” Participant 5, Healthcare trainee, Woman. |
| - Impact on mental wellbeing | “I didn’t realise until I started the app (MYARKEO). That I was in pain every day, which was really interesting. Then I reflected on that. I started to get up and move more in […03:08] and also, I think it helped me kind of understand that really I enjoy working in an office more because I found when I was working in the office more my mental health was slightly better.” Participant 3, Primary care counsellor, Non-binary |
| Enablers of engagement |  |
| - Increased awareness | “I think the aspect I liked most was solved holding myself accountable. I felt like I’d be at the end of the day, with how many caffeinated drinks I’d had or how many hours of work I had. So, I guess that made me mindful of what I was doing throughout the day. So, I felt like I was holding myself accountable, and I think that was probably the most enjoyable aspect.” Participant 12, Practitioner psychologist, Man |
| - Personal strategies | “It was on my home page so I tended to see it there, but I think the future I would set, and I would get the notifications to remind me more.” Participant 9, Nurse, Woman |
| - External motivators | “It was quick and easy to input. And also, a couple of times you sent reminders at the weekend and I didn’t forget most of the time.” Participant 1, Nurse, Woman |
| Barriers to engagement |  |
| - Personal challenges | “There were a few days where I just completely forgot it was just so hectic that. I didn’t get to log, but I did try my upmost best to do every day.” Participant 11, Healthcare trainee, Woman |
| - Motivational challenges | “There is a simple formula or algorithm to show my situation, or my problem based on my answers. I think that your application provides to observe myself, my problem, or my emotional point of view. But I don’t think it is offering any solution or answer to solve them.” Participant 8 Healthcare trainee, Woman |
| - Technical challenges | “A couple of times it didn’t work. So, there’s a couple of times, I think, when it was on about where you at work. If not, you weren’t at work. Sometimes, it asks you for a reason, and sometimes, it doesn’t. And then I think there was one day when it just wouldn’t load at all. So, I just didn’t enter anything.” Participant 13, Nurse, Woman |
| Suggested intervention improvements |  |
| - Work-oriented recommendations | “Maybe have some things that are in there that may be tailored towards people in healthcare. And make it a little bit more personal. NHS and people working in private hospitals as well. Don’t leave them out. But then yeah, just generally, I suppose, maybe recognised their stress levels, and are they drinking and how are they hydrated? That’s one of the issues as well. That’s often causes people to be rundown and ill. And you know, are they getting that time off appropriately to recover. Yeah, maybe make it more personal to them.” Participant 11, Healthcare trainee, Woman |
| - Content improvements | “I think having goals or targets that you can set. So, for example, because the effects of stopping caffeine won’t be immediate. You could maybe do a target for yourself somehow incorporated with the app. So, two weeks no caffeine. From harden your caffeine use. So, for example, once the app has some data on your caffeine news that can tell you what your average number of cups are, and then how you can cut that by half over two weeks and then see its effect on anxiety with graphs and stuff. I guess setting targets and then doing that target for a couple of weeks, and then the app could show you how your low mood in the previous two weeks was compared to the current two weeks.” Participant 2, Physiotherapist, Woman |
| - Design improvements | “If it was maybe a bit brighter as well. You know, so it stands out a bit more because, you know, if you’ve got an iPhone, then you have lots of applications. And even though it literally was sitting on its own. Just had to keep remembering what it was. So maybe it was a bit brighter or had a health, something healthy in it, or I don't know. Something that might trigger it a little bit more.” Participant 13, Nurse, Woman |
