# Supplementary material for: Supporting mental well-being of healthcare workers using a mobile app: A mixed-methods feasibility study
Source: PLoS One. 2026 Jan 16;21(1):e0341055. doi: 10.1371/journal.pone.0341055 (PMC12810850; doi:10.1371/journal.pone.0341055)
Supplement: S1 Text — (DOCX) [file pone.0341055.s007.docx]

**S1 Text.** Interview topic guide

**A mobile app-based intervention to promote mental wellbeing in healthcare workers/trainees**

**INTERVIEW TOPIC GUIDE**

**Introduction:**

Hi _____________, my name is ______________________Thank you for agreeing to speak with me today about our project, I am the member of the research team conducting the study about mental fitness improvement in healthcare workers through a mobile app, MYARKEO. This is an evaluation of users’ experiences of using the mobile app.

As outlined in the accompanying information, we would like to record this interview, with your permission, so that we can use anonymised quotes from this interview when we produce summaries of our findings. This will help us to clarify nurses’ views of the mobile app. Your comments are confidential which means that at no point will you be identifiable in our data analysis and report. We will anonymise any content that we use in publications.

Before we start, do you have any questions for me?

Are you happy for this interview to be recorded? Thank you.

Questions

1- Could you start by telling me a bit about your overall experience of using the MYARKEO app?

[prompts: What are your initial thoughts about the use? How easy was it to use the app? What you like most about the app?]

2- Did the app have any impact on your wellbeing or mental health? If so in what ways? If not Why?

[prompts: What makes you say that? Can you tell me more about that? Was there anything else you noticed? How did the app affect your mental well-being? Did it help you to improve your daily mood?]

3- Did you make any changes to your behaviour while using the app? [If yes] Could you tell me more about these changes? [If no] Are there any reasons why you did not make changes?

[prompts ask for specific behaviour change and impacts on the life, ask for the reason why the app did not lead to behaviour change, e.g., individual reasons or organisational reasons or other]

4- What helped to ensure your consistent use of the app? [What was most useful about the app? What was least useful?].

[prompts ask for specific reasons]

5- Did you experience any issues while using the app or problems using it?

[prompts easy to use, the app content, accessibility, articles on the app about wellbeing, any issues with accessibility, technical issues etc.]

6- Did you stop using the app at any point? If so, could you tell me why?

[prompts ask for any specific reasons. If you stop using the app, what would be helpful for you to continue using the app again?]

7- Do you think that the app might be useful to support mental wellbeing of healthcare workers? In what ways?

[prompts: In what aspects could the app be helpful most? Improving daily mood, behaviour change or improving symptoms such as stress level, anxiety etc.]

8- How could the app be improved for use with healthcare workers?

[prompts ask for app content, articles, interface, language, etc.]

Close: Is there anything else you would like to share with me today?

Thank you for sharing your experiences with me today. It has been interesting to hear your views on this mobile app. [if discussion raised difficult emotions - I am aware that this discussion might have raised some difficult emotions, so I just want to check that you are feeling okay now?]

In terms of what happens next, we are speaking with other nurses about their views of this app. We will write up the analysis of these interviews (anonymised) and we intend to publish our project findings in an academic journal.

Thanks again for taking the time to speak with me today.
