# Supplementary material for: Supporting mental well-being of healthcare workers using a mobile app: A mixed-methods feasibility study
Source: PLoS One. 2026 Jan 16;21(1):e0341055. doi: 10.1371/journal.pone.0341055 (PMC12810850; doi:10.1371/journal.pone.0341055)
Supplement: Attachment — Submitted filename: Response to reviewers.docx [file pone.0341055.s010.docx]

Dear Editor,

Thank you for the opportunity to revise and resubmit our article.

A point-by-point response is provided below.

We look forward to hearing from you in due course.

Kind regards,

Prof. Holly Blake (corresponding author).

| **Comments** | **Revisions** |
| --- | --- |
| **Reviewer 1** |  |
| This article aims to assess the feasibility of a self-monitoring mobile app called MYARKEO, to improve mental well-being among healthcare workers and healthcare trainees in the United Kingdom (UK).  First, in the introduction section, authors mentioned "Despite the increasing use of apps for wellbeing, the feasibility, usability and acceptability of such tools has not been investigated with healthcare workers or healthcare trainees.” This section should be rewritten because the author(s) only need to clearly point out the theoretical basis and the reason of using these theories in this part. | Many thanks for this comment. We have checked the introduction and revised the flow of the paragraphs.  We have added a section into the end of paragraph 3 (line 61) in which we mention mobile app-based mental wellbeing interventions. This improved the flow. |
| Second, the literature review section is deficient in several key areas. It fails to sufficiently address recent scholarly works pertinent to the topic in the following respects:  1. “Chatbot as an emergency exist: Mediated empathy for resilience via human-AI interaction during the COVID-19 pandemic”  2. “Disentangling composite influences of social connectivity and system interactivity on continuance intention in mobile short video applications: The pivotal moderation of user-perceived benefits”  3. “Determining influence of service quality on user identification, belongingness, and satisfaction on mobile social media: Insight from emotional attachment perspective”  4. “How multidimensional benefits determine cumulative satisfaction and eWOM engagement on mobile social media: Reconciling motivation and expectation disconfirmation perspectives”  5.“Struggling or Shifting? Deciphering potential influences of cyberbullying perpetration and communication overload on mobile app switching intention through social cognitive approach”  6. “Determining the influence of depressive mood and self-disclosure on problematic mobile app use and declined educational attainment: Insight from stressor-strain-outcome perspective”  7．“Deciphering dynamic effects of mobile app addiction, privacy concern and cognitive overload on subjective well-being and academic expectancy: The pivotal function of perceived technostress” | Thank you for sending details of these 7 studies, all of which have a single author in common.  These studies are not directly relevant to our paper. One is about an AI-powered Chatbot, the others relate to a range of issues as mobile social media, cyberbullying, and technostress.  We have discussed these papers as a research team and agreed that they are not directly relevant to the topic area of this paper and would detract from its focus. |
| Third, more details about the method are needed to add in this current article. Language needs polishing: many sentences are not very smooth. | Thank you for this comment. We have made some minor revisions to sentences throughout the paper (particularly the methods) to improve the flow. We noticed inconsistency in the presentation of well-being (well-being or wellbeing) and this has been corrected throughout.  It is not clear what further methodological details are required by this reviewer – we have already included all relevant details for this study. One change we did make is to move the signposting to the interview topic guide (S1 Text) from the results section into the methods. |
| Fourth, according to the results, some findings were proposed. Combined with related work, the authors should accentuate their contributions in the conclusion section of this paper. | Thank you for the opportunity to further accentuate our findings. We have endeavoured to do this with a re-draft of text in the conclusion section. |
| Finally, while the paper is well-structured, there are still some issues with the clarity of expression and language, undermining its readability. Thus, the authors should ensure that a revised version of the manuscript is thoroughly proofread. | Thank you for this comment. We have re-read the manuscript and made minor revisions to the language used throughout. |
| **Reviewer 2** |  |
| 1. Methodological Considerations  Technical Issue Specificity: In Theme 3: Barriers to engagement – Technical Challenges, the mention of “software bugs” is too general. Since the app is central to the study, it would be helpful to describe the types of bugs encountered and their impact. Login issues on Android and the lack of a password reset feature appear to have contributed to attrition. Given that the app was updated during the study, please clarify whether these problems were resolved and why participants were not encouraged to update, as updates typically address such usability concerns.  Engagement Calculation: The reported 64.41% engagement rate (514/789 days) lacks detail. Please clarify how this was calculated over the 6-week period, including definitions of engagement and the denominator used.  Tracking Frequency: Please specify how often participants used the app each day. This data is often available from app analytics. Encouraging more frequent use may have yielded richer insights, as prior research (e.g., Myin-Germeys et al., 2009) shows the importance of capturing intra-day fluctuations in mental health using Ecological Momentary Assessment (EMA). | Many thanks for these helpful comments. In Theme 3, “unresponsive questions, delayed saving of data, and daily scores not loading” are the minor bugs. The next sentence (line 391) was revised, those bugs (occurred a few times) were gone after restarting the app. These bugs may also occur based on the phone quality.  There is one major technical issue with Android phones which is mentioned in line 231. This issue occurred during the recruitment period. We contacted the app developer, but unfortunately the issue could not be resolved. Since we had already reached our target number of participants, recruitment stopped.  Mobile engagement means frequency of mobile app use, which is someone entering the app and answering the daily questions. We had 19 participants in the intervention group. It was expected all participants would use the app every day, total of 42 days. So, 19x42= 798 which is the total days. However, some participants used the app every day, but some only used it for a few days. When somebody used the app, the app saved the day and their daily score. Based on these records, we calculated the total actual use which was 514. Then, we divided 514/798 =64.4% that is the daily engagement rate. In the method section line 186-189, a sentence was added to describe how the engagement rate was calculated.  While re-checking the numbers, we noticed that there was a mistake relating to the total number. It is 798 but was mistakenly reported as 789. This has been corrected and all other numbers have been checked and are correct.  Many thanks for this suggestion. However, since participant were encourage to use the app once per day for daily monitoring, we did not observe more than one use in a day and would not expect to in this particular study. |
| 2. Inconsistencies  Usability Ratings: The usability section rates ease of use as “excellent,” yet interface satisfaction and usefulness are rated “poor.” This contradiction should be clarified.  Participant Feedback vs. Quantitative Ratings: In Theme 1, participants are quoted as praising the app’s design and ease of use, yet this conflicts with the MAUQ ratings. Clarifying this discrepancy would improve interpretive consistency.  Dropout and Well-being Scores: The "assessment of mental well-being, depression, stress and anxiety" section mentions that participants who dropped out had higher baseline well-being scores than those who completed the study. This pattern appears to undermine the conclusion that the app supports positive behavior change or improved well-being, and should be discussed. | Thank you for this comment. This MAUQ included 18 questions, which were divided into three groups: ease of use (the first five questions), interface and satisfaction (the following seven questions), and usefulness (the last six questions). Participants expressed different views on these three groups.  The findings from the MAUQ are consistent with the qualitative findings. In the themes, the participants like the ease of use of the app. However, they indicate some cons about interface and satisfaction such as “masculine interface” (in Theme 4, design improvements) and perceived lack of usefulness (in Theme 1, Impacts on mental wellbeing). The MAUQ total score reflects these differences in views as its “good” in between “excellent” and “poor”.  Many thanks for this thoughtful comment. However, high well-being score is better mental wellbeing based on the interpretation of the scoring of WEMWBS. In the intervention group, post-intervention mental well-being score is slightly lower than baseline mental-wellbeing score. It is also lower than the control group scores. So, this may mean that the app may not show significant improvement in the well-being scores. We have reflected on this in the discussion (line 446-459), although, as we have now added to the text, it is not the intention of a feasibility study to examine / determine effectiveness of an intervention. Small sample size, and higher well-being scores in drop-outs may impact on these differences. |
| 3. Reporting Clarity  Repetition: The phrase “expectation of immediate benefits” is repeated in Theme 3 – Motivational Challenges and could be consolidated.  Comparative Claims: The claim that engagement was higher than in similar studies needs supporting references to validate the comparison.  The study explores an important area of interest. Addressing the issues above will improve the manuscript’s clarity, methodological transparency, and alignment between data and conclusions. | Yes, thank you for noticing this. The repetition has now been removed.  We appreciated the suggestion to cite references for our comparison with other studies relating to engagement. We have already cited two articles in which the sample is a healthcare population.  Knill K, Warren B, Melnyk B, Thrane SE. Burnout and well-being: Evaluating perceptions in bone marrow transplantation nurses using a mindfulness application. Clin J Oncol Nurs. 2021;25: 547–554. doi:10.1188/21.CJON.547-554  Orosa-Duarte Á, Mediavilla R, Muñoz-Sanjose A, Palao Á, Garde J, López-Herrero V, et al. Mindfulness-based mobile app reduces anxiety and increases self-compassion in healthcare students: A randomised controlled trial. Med Teach. 2021;43: 686–693. doi:10.1080/0142159X.2021.1887835    To further address your suggestion, we have now added a recent meta-analysis of persuasive design, engagement, and efficacy in 92 RCTs of mental health apps to support our argument that our engagement rate is higher than overall daily app use.  Valentine L, Hinton JDX, Bajaj K, Boyd L, O’Sullivan S, Sorenson RP, et al. A meta-analysis of persuasive design, engagement, and efficacy in 92 RCTs of mental health apps. NPJ Digit Med. 2025;8: 229. doi:10.1038/s41746-025-01567-5  Many thanks for your supportive comments which we believe have improved the manuscript quality. |

**Journal Requirements:**

When submitting your revision, we need you to address these additional requirements.

1. Please ensure that your manuscript meets PLOS ONE's style requirements, including those for file naming. The PLOS ONE style templates can be found at

<https://journals.plos.org/plosone/s/file?id=wjVg/PLOSOne_formatting_sample_main_body.pdf>

<https://journals.plos.org/plosone/s/file?id=ba62/PLOSOne_formatting_sample_title_authors_affiliations.pdf>

The manuscript aligns with journal requirements.

2. We note that the grant information you provided in the ‘Funding Information’ and ‘Financial Disclosure’ sections do not match.

When you resubmit, please ensure that you provide the correct grant numbers for the awards you received for your study in the ‘Funding Information’ section.

This has been actioned to ensure that they match.

3 .If the reviewer comments include a recommendation to cite specific previously published works, please review and evaluate these publications to determine whether they are relevant and should be cited. There is no requirement to cite these works unless the editor has indicated otherwise.

We do not believe that any of the recommended articles (Reviewer 1) are relevant to our study and therefore we have chosen not to include them.
