# Supplementary material for: Supporting mental well-being of healthcare workers using a mobile app: A mixed-methods feasibility study
Source: PLoS One. 2026 Jan 16;21(1):e0341055. doi: 10.1371/journal.pone.0341055 (PMC12810850; doi:10.1371/journal.pone.0341055)
Supplement: Attachment — Submitted filename: Response_to_reviewers_auresp_3.docx [file pone.0341055.s012.docx]

17 December 2025

Dear Editor and Reviewers,

PONE-D-25-19125R2

Supporting mental well-being of healthcare workers using a mobile app: a mixed-methods feasibility study

We have received a request for minor revisions to the above paper. However, there do not seem to be any revisions to address.

We have responded to the points made below which confirm this. Consequently, no changes have been made to the documents submitted last time.

**If you would like to make changes to your financial disclosure, please include your updated statement in your cover letter**

We have no changes to make, the information already included is correct, although we have included it again in our cover letter.

**Comments to the Author**

**1. If the authors have adequately addressed your comments raised in a previous round of review and you feel that this manuscript is now acceptable for publication, you may indicate that here to bypass the “Comments to the Author” section, enter your conflict of interest statement in the “Confidential to Editor” section, and submit your "Accept" recommendation.**

**Reviewer #2: All comments have been addressed**

Thank you for this positive comment.

**2. Is the manuscript technically sound, and do the data support the conclusions?

The manuscript must describe a technically sound piece of scientific research with data that supports the conclusions. Experiments must have been conducted rigorously, with appropriate controls, replication, and sample sizes. The conclusions must be drawn appropriately based on the data presented.**

**Reviewer #2: Yes**

Thank you for confirming our manuscript is technically sound.

**3. Has the statistical analysis been performed appropriately and rigorously?**

**Reviewer #2: Yes**

Thank you confirming the statistical analysis has been performed appropriately and rigorously.

**4. Have the authors made all data underlying the findings in their manuscript fully available?

The** [**PLOS Data policy**](http://www.plosone.org/static/policies.action#sharing) **requires authors to make all data underlying the findings described in their manuscript fully available without restriction, with rare exception (please refer to the Data Availability Statement in the manuscript PDF file). The data should be provided as part of the manuscript or its supporting information, or deposited to a public repository. For example, in addition to summary statistics, the data points behind means, medians and variance measures should be available. If there are restrictions on publicly sharing data—e.g. participant privacy or use of data from a third party—those must be specified.**

**Reviewer #2: Yes**

Thank you for confirming our data are fully available in a research data management repository.

**5. Is the manuscript presented in an intelligible fashion and written in standard English?

PLOS ONE does not copyedit accepted manuscripts, so the language in submitted articles must be clear, correct, and unambiguous. Any typographical or grammatical errors should be corrected at revision, so please note any specific errors here.**

**Reviewer #2: Yes**

Thank you for confirming that our manuscript is written in an intelligible fashion and standard English.

**6. Review Comments to the Author

Please use the space provided to explain your answers to the questions above. You may also include additional comments for the author, including concerns about dual publication, research ethics, or publication ethics. (Please upload your review as an attachment if it exceeds 20,000 characters)**

**Reviewer #2: All comments have been addressed in the latest submission. The Android problem specifically is also added.**

Thank you for confirming that all comments were addressed in the previous submission.

**To ensure your figures meet our technical requirements, please review our figure guidelines: https://journals.plos.org/plosone/s/figures**

We have checked and our figures do meet technical requirements.
